# Flexible and Robust Functionalized Boron Nitride/Poly(*p*-Phenylene Benzobisoxazole) Nanocomposite Paper with High Thermal Conductivity and Outstanding Electrical Insulation

**DOI:** 10.1007/s40820-023-01257-5

**Published:** 2023-11-30

**Authors:** Lin Tang, Kunpeng Ruan, Xi Liu, Yusheng Tang, Yali Zhang, Junwei Gu

**Affiliations:** 1 Chongqing Key Laboratory of Green Synthesis and Applications, College of Chemistry, Chongqing Normal University, Chongqing, 401331 People’s Republic of China; 2 Shaanxi Key Laboratory of Macromolecular Science and Technology, School of Chemistry and Chemical Engineering, Northwestern Polytechnical University, Xi’an, 710072 People’s Republic of China

**Keywords:** Poly(*p*-phenylene-2,6-benzobisoxazole) nanofiber, Boron nitride, Thermal conductivity, Electrical insulation

## Abstract

With the rapid development of 5G information technology, thermal conductivity/dissipation problems of highly integrated electronic devices and electrical equipment are becoming prominent. In this work, “high-temperature solid-phase & diazonium salt decomposition” method is carried out to prepare benzidine-functionalized boron nitride (*m*-BN). Subsequently, *m*-BN/poly(*p*-phenylene benzobisoxazole) nanofiber (PNF) nanocomposite paper with nacre-mimetic layered structures is prepared via sol–gel film transformation approach. The obtained *m*-BN/PNF nanocomposite paper with 50 wt% *m*-BN presents excellent thermal conductivity, incredible electrical insulation, outstanding mechanical properties and thermal stability, due to the construction of extensive hydrogen bonds and π–π interactions between *m*-BN and PNF, and stable nacre-mimetic layered structures. Its *λ*_∥_ and *λ*_⊥_ are 9.68 and 0.84 W m^−1^ K^−1^, and the volume resistivity and breakdown strength are as high as 2.3 × 10^15^ Ω cm and 324.2 kV mm^−1^, respectively. Besides, it also presents extremely high tensile strength of 193.6 MPa and thermal decomposition temperature of 640 °C, showing a broad application prospect in high-end thermal management fields such as electronic devices and electrical equipment.

**Supplementary Information:**

The online version contains supplementary material available at 10.1007/s40820-023-01257-5.

## Highlights

- *m*-BN/PNF nanocomposite paper with nacre-mimetic layered structures prepared via sol–gel film transformation approach presents excellent thermal conductivity, incredible electrical insulation, outstanding mechanical property and thermal stability.
- When the mass fraction of *m*-BN is 50 wt%, *m*-BN/PNF nanocomposite paper exhibits excellent thermal conductivity and electrical insulation. The *λ*_∥_ and *λ*_⊥_ are 9.68 and 0.84 W m^−1^ K^−1^, and the volume resistivity and breakdown strength are as high as 2.3 × 10^15^ Ω cm and 324.2 kV mm^−1^, respectively.
- The *m*-BN/PNF nanocomposite paper with 50 wt% *m*-BN also presents outstanding mechanical properties (tensile strength of 193.6 MPa) and thermal stability (thermal decomposition temperature of 640 °C).

**Supplementary Information:**

The online version contains supplementary material available at 10.1007/s40820-023-01257-5.

## Graphical abstract

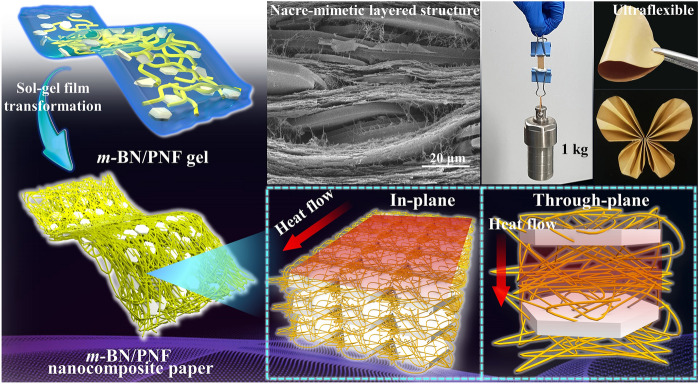

## Introduction

Thermally conductive polymer-based composite paper has attracted widespread attention in the fields of lithium batteries, capacitors and integrated circuits [1–4], due to the advantages of high strength, high thermal conductivity and excellent designability, etc*.* With the rapid development toward miniaturization and integration, as well as increasing power density, the build-up of heat inside electronic devices and electrical equipment is getting serious, which puts forward higher requirements for the thermal conductivity and heat resistance of thermally conductive polymer-based composite paper [5–7]. Moreover, in order to avoid the formation of short-circuit currents between microelectrical components and the mutual interference of signals, the polymer-based composite paper should possess outstanding electrical insulation to meet the application in practical electronic engineering [8–10]. Although thermally conductive polymer matrix (polytetrafluoroethylene, polyimide, aramid and cellulose nanofibers, etc*.*) composite paper has been widely used in industry owing to their low-cost and simple processing technology, the intrinsically poor heat resistance, or poor mechanical properties, or low thermal conductivity limits their application and no longer guarantees the stability and reliability in the fields of thermal management for high-end electronics and electrical appliances [11–13].

Among the known organic fibers, poly(*p*-phenylene-2,6-benzobisoxazole) (PBO) fibers present the highest thermal decomposition temperature (650 °C), the best tensile strength (5.8 GPa) and tensile modulus (280 GPa) and have been hailed as the super fiber of the twenty-first century [14–16]. Recent studies exhibit that PBO nanofibers (PNF) obtained by organic acid stripping can retain the excellent mechanical properties and heat resistances of PBO fibers [17–20]. In addition, their interior contains highly oriented molecular chains and original crystallinity, showing better thermal conductivity than ordinary polymer matrix, which shows a broad application prospect in the field of thermally conductive polymer-based composite paper. Li et al. [21] reported a series of fluorinated graphene (FG)-based nanocomposite films containing robust PNF network structures via unique sol–gel film conversion method. The nanocomposite film with 40 wt% FG possessed high in-plane thermal conductivity coefficient (*λ*_∥_, 12.3 W m^−1^ K^−1^), 392% higher than that of pure PNF paper (2.50 W m^−1^ K^−1^). Zhao et al. [22] introduced boron nitride (BN) and MXene sequentially into the PNF networks to prepare PNF/BN/MXene composite paper via gel microparticle-mediated ordered assembly process with the aid of vacuum-assisted filtration. When the amounts of BN and MXene were 29.2 and 41.7 wt%, respectively, the *λ*_∥_ of PNF/BN/MXene composite paper was 26.10 W m^−1^ K^−1^, significantly higher than that of pure PNF paper (2.92 W m^−1^ K^−1^). Nevertheless, the introduction of functional fillers (such as graphene and MXene) can drastically decrease the electrical insulation of PNF-based composite paper [23–26], limiting its broader application in electrical and electronic fields. It remains a great challenge to develop high-performance PNF-based composite paper with excellent thermal conductivity and electrical insulation.

BN presents excellent thermal conductivity, electrical insulation and heat resistance, showing good application prospects in the thermal management fields of electronic and electrical [27–31]. Yu et al. [32] prepared BN/epoxy composites by blending BN with epoxy resin. When the amount of BN was 11.9 wt%, the thermal conductivity coefficient (*λ*) of BN/epoxy composites increased from 0.21 to 0.51 W m^−1^ K^−1^, and the breakdown strength increased from 40.9 to 58.6 kV mm^−1^. Yang et al. [33] reported the facile and scalable approach to fabricate elastomeric silicone rubber (SiR)/graphene nanoparticles (GNPs)/BN composites with an alternating multilayer structure, achieving high *λ* of 8.45 W m^−1^ K^−1^ and excellent electrical insulation properties (volume resistivity of about 10^13^ Ω cm and breakdown strength of 5.33 kV mm^−1^). However, the enhancement of thermal conductivity was limited due to the high interfacial thermal resistance between BN and BN fillers, as well as BN and polymer matrix [34–36]. In our previous work, Gu et al. [37] calculated the interfacial thermal resistance between fillers and polymer matrix based on the modified Hashin–Shtrikman model and effective medium theory, revealing that the surface-functionalized fillers could further improve the *λ* of thermally conductive composites with the same amount of fillers. Therefore, the surface functionalization of BN is the key factor for reducing interfacial thermal resistance and further improving the *λ* of corresponding thermally conductive composites.

To our knowledge, the surface functionalization methods of BN fillers are mainly classified into non-covalent modification and covalent modification [38–40]. The former utilizes van der Waals force and electrostatic adsorption, etc*.*, to coat the surface of BN with a layer of organic matter [41–43]. However, the functionalized BN prepared by the non-covalent modification is unstable. Covalent modification is to destroy B–N bond on the surface of BN by plasma, ultrasonic treatment, strong acid, and strong base to form stable hydroxyl and amino groups [44–46]. Kim et al. [47] carried out ultrasonic treatment of BN and then adopted (hexadecyl)trimethoxysilane to modify the surface of BN (C16-BN). C16-BN/epoxy composites with 20 wt% C16-BN presented excellent *λ* (3.49 W m^−1^ K^−1^), 45.4% higher than that of 20 wt% BN/epoxy composites (2.40 W m^−1^ K^−1^). Zhang et al. [48] adopted KOH/NaOH to treat BN by the high-temperature solid-phase method to obtain BN-OH and subsequently blended with polystyrene (PS) to prepare BN-OH/PS composites. The native structure of BN-OH was not damaged. When the amount of BN-OH was 12 wt%, the BN-OH/PS composites presented excellent thermal conductivity with *λ* of 1.13 W m^−1^ K^−1^, 52.7% higher than that of 12 wt% BN/PS composites (0.74 W m^−1^ K^−1^).

In this work, PBO fibers are stripped in the methanesulfonic acid (MSA)/trifluoroacetic acid (TFA) solution to obtain PNF. Benzidine is performed to functionalize the surface of BN (*m*-BN), followed by blending with PNF to prepare the *m*-BN/PNF nanocomposite paper via sol–gel film transformation approach. X-ray diffractometer (XRD), X-ray photoelectron spectroscopy (XPS), scanning electron microscope (SEM) and transmission electron microscope (TEM) are utilized to analyze and characterize the surface elements, crystal structures and micromorphologies of *m*-BN and PNF. On this basis, the functionalization of *m*-BN and its amount influencing on thermal conductivities, electrical insulation and mechanical properties of the *m*-BN/PNF nanocomposite paper are analyzed.

## Experimental Section

### Surface Functionalization of BN

Benzidine is performed to functionalize the surface of BN (*m*-BN) by “high-temperature solid-phase & diazonium salt decomposition” method. The B–N on the surface of BN can be destroyed to generate hydroxyl and amino groups (HO-BN) via high-temperature solid-phase method [49] at concentrated alkaline environment. The high activity of benzidine carbocation produced by decomposition of diazonium salt is utilized to graft benzidine onto the surface of HO-BN (*m*-BN). The surface functionalization mechanism of *m*-BN is shown in Fig. S1, and the specific process is as follows.

One gram of BN, 1.5 g of sodium hydroxide and 1.5 g of potassium hydroxide were ground into powder, reacting in a hydrothermal reactor at 180 °C for 5 h. Subsequently, the reactants were dissolved in distilled water and washed for 2–3 times until the solution was neutral, followed by drying at 60 °C for 24 h to obtain HO-BN. Next, 3.7 g of benzidine was dissolved in distilled water, stirred in an ice bath, and an appropriate amount of hydrochloric acid was added to form amaranth suspension. Then, 1.4 g of sodium nitrite was slowly added into the above solution to form a diazonium chloride salt solution. Finally, 0.5 g of HO-BN was added into the diazonium chloride salt solution, 2.0 g of iron powder and an appropriate amount of hydrochloric acid were added sequentially, and the reaction was carried out at ice bath environment for 2 h. The reactants were washed with distilled water and methanol to remove excess diazonium chloride salts, followed by drying at 60 °C for 24 h to obtain *m*-BN.

### Fabrication of the *m*-BN/PNF Nanocomposite Paper

**Fig. 1:**
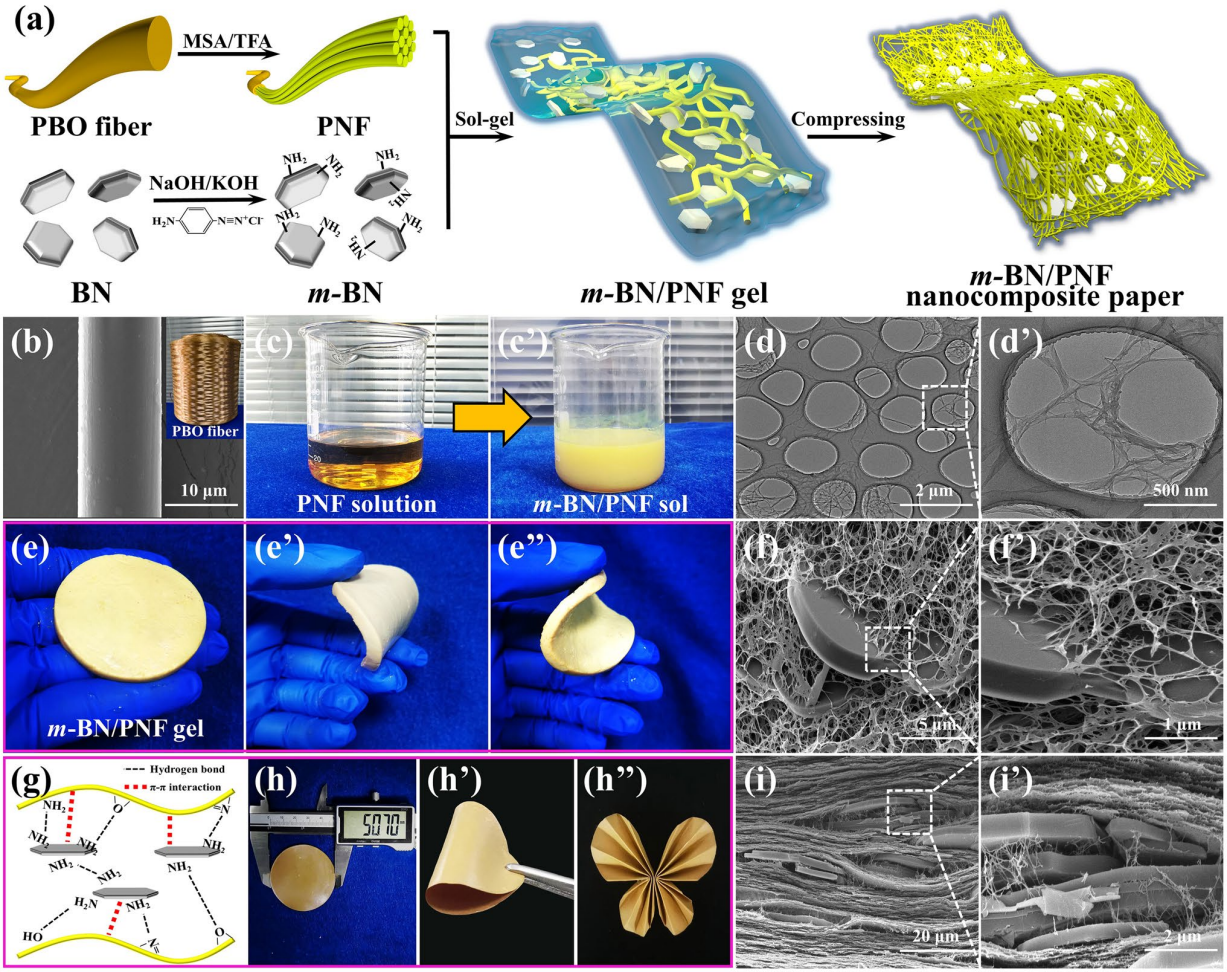
Schematic diagram of the preparation process for *m*-BN/PNF nanocomposite paper (**a**); optical photograph and SEM image of PBO fibers (**b**); the process of converting PNF solution into *m*-BN/PNF sol (**c**, **c′**); TEM images of PNF (**d**, **d′**); optical photographs of *m*-BN/PNF gel with certain flexibility (**e**, **e″**); SEM images showing the inside of *m*-BN/PNF gel (**f**, **f′**); schematic diagram of the interaction mechanism between PNF and *m*-BN (**g**); optical photographs of *m*-BN/PNF nanocomposite paper showing excellent flexibility and foldability (**h**, **h″**); cross-sectional SEM images of *m*-BN/PNF nanocomposite paper (**i**, **i′**)

PBO fibers were added into the MSA/TFA solution with a volume ratio of 1:1, followed by stirring for 24 h to form PNF dispersion. Appropriate amount of *m*-BN and 1.5 g of Na_2_SO_4_ were added into 30 g of PNF dispersion (0.1 wt%) and magnetically stirred for 2 h. Subsequently, 10 g of PNF dispersion (1 wt%) was continued to be added, and the *m*-BN/PNF acid sol was obtained by high-speed homogeneous stirring. The acid sol was poured into a culture dish and stood for 24 h to obtain *m*-BN/PNF acid gel. Then, the *m*-BN/PNF acid gel was soaked in distilled water for multiple solvent exchange, to obtain the *m*-BN/PNF hydrogel, which was then compressed and dried to obtain *m*-BN/PNF nanocomposite paper. The schematic diagram of the preparation process is shown in Fig. 1a. A series of *m*-BN/PNF-X nanocomposite paper was fabricated by changing the mass ratio of *m*-BN to PNF (X represented the mass fraction of *m*-BN, which were 10, 20, 30, 40 and 50, respectively). In addition, a series of BN/PNF-X composite paper was prepared by the same method for comparative analyses.

## Results and Discussion

### Structures and Morphologies of *m*-BN/PNF Nanocomposite Paper

Figure 1b shows the SEM image and optical photograph of PBO fibers. PBO fibers have been gradually exfoliated in the mixed acid of MSA/TFA, becoming brown PNF dispersion (Fig. 1c). Figure 1d, d′ shows the TEM images of high-aspect-ratio PNF with diameter ranging from 10 to 40 nm. Nitrogen and oxygen atoms on PBO molecular chains are protonated by the mixed acid, which increases the electrostatic repulsion between the molecular chains, resulting in the gradual stripping of PBO fibers to PNF. Meanwhile, some oxazole rings on the PBO molecular chains are broken to generate hydroxyl and amino groups (Fig. S2).

The yellow *m*-BN/PNF acid sol (Fig. 1c′) is formed by adding *m*-BN and Na_2_SO_4_ into the PNF dispersion. The acid sol has been poured into a culture dish and stood for 24 h to obtain *m*-BN/PNF acid gel with certain flexibility (Figs. S3a, a′ and 1e, e″). On the contrary, the pristine PNF dispersion shows no significant changes after standing for 24 h. As can be seen in Fig. 1f, f′, *m*-BN and PNF form a stable three-dimensional (3D) crosslinked network structures, and PNF is tightly attached onto the surface of *m*-BN. The network is created because the SO_4_^2−^ shields the positive charge on the surface of PNF, which suppresses electrostatic exclusion and enhances the π–π interactions between PNF, leading to crosslinking for gelation (Fig. S4a, a′). In addition, amino groups and biphenyls on the surface of *m*-BN form strong hydrogen bonds and π–π interactions with PNF (Fig. 1g), causing PNF to adhere closely onto the surface of *m*-BN. However, original BN forms obvious defects in 3D network structure of PNF owing to weak interaction (Fig. S4b, b′).

The* m*-BN/PNF nanocomposite paper presents excellent flexibility and folding resistance, with no obvious damage after being bent for 180 degrees or folded into a bow (Fig. 1h–h″). As shown in Fig. 1i, i′, based on the hydrogen bonds and π–π interactions, *m*-BN/PNF nanocomposite paper displays an orderly arrangement of *m*-BN in the in-plane direction. A large amount of PNF is tightly stacked and interconnected between the *m*-BN in the through-plane direction, resulting in stable nacre-mimetic layered structures inside the nanocomposite paper (Fig. S5). Besides, *m*-BN is uniformly distributed on the surface of the *m*-BN/PNF nanocomposite paper, and PNF on the surface is interlaced with each other to form a porous nanofiber network structure (Fig. S6).

**Fig. 2:**
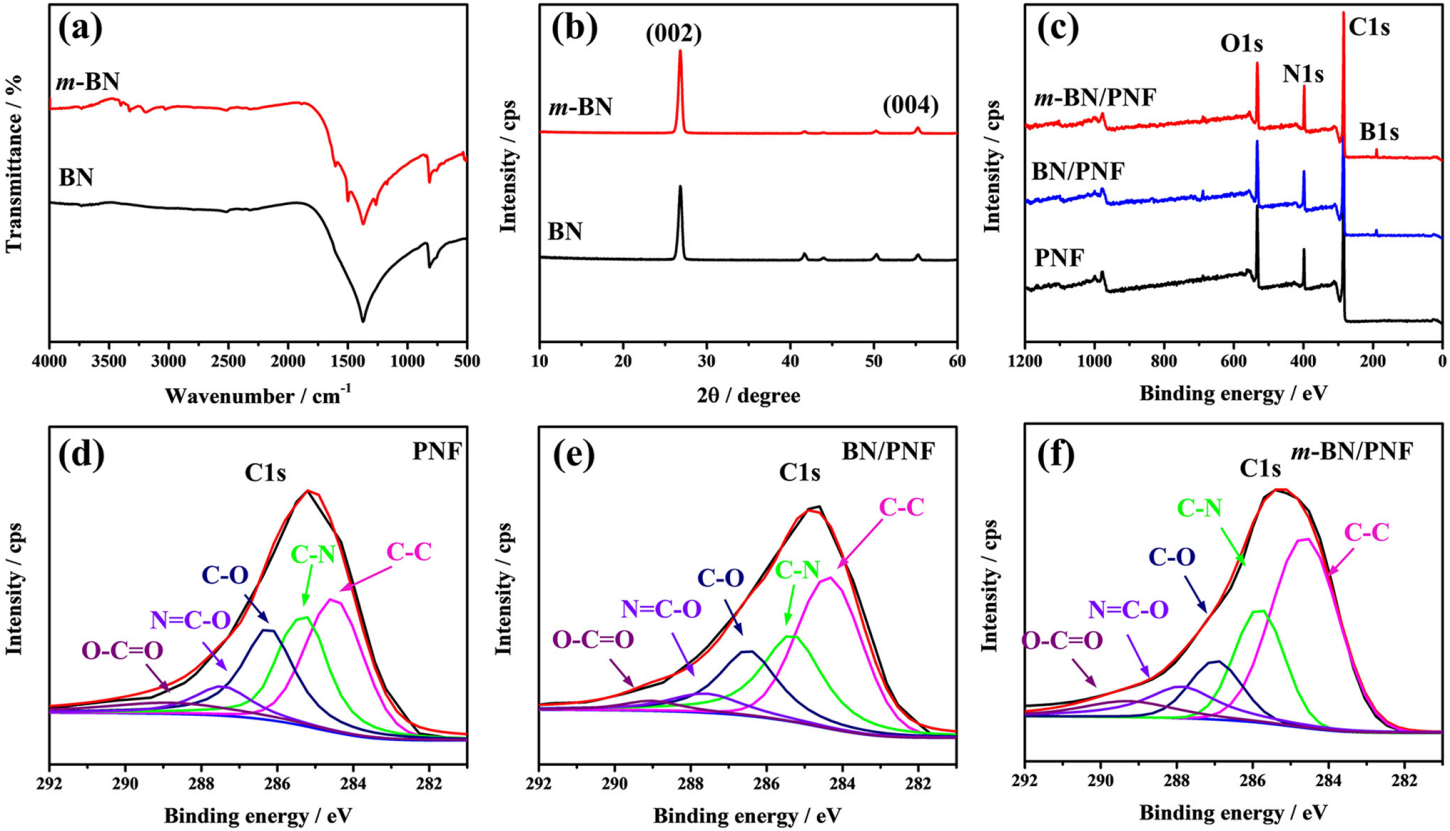
FTIR (**a**) and XRD (**b**) spectra of *m*-BN and BN; XPS wide-scan spectra (**c**) and high-resolution C 1*s* XPS spectra (**d–f**) of PNF paper, BN/PNF and *m*-BN/PNF nanocomposite paper

Figure 2a, b shows Fourier transformed infrared (FTIR, a) and XRD (b) spectra of the BN before and after functionalization. BN shows obvious absorption peaks at 811 and 1376 cm^−1^ (Fig. 2a), corresponding to the stretching and bending vibration absorption of B–N, respectively [50, 51]. Compared with BN, *m*-BN appears new characteristic absorption peaks at 1608 and 1500 cm^−1^, mainly attributed to the stretching vibration peak of biphenyls. Characteristic absorption peak corresponding to the amino group appears at 3400 cm^−1^, which proves that benzidine is successfully grafted on the surface of BN. As can be seen from the high-resolution XPS spectra of N1s before and after BN functionalization (Fig. S7a, b), in addition to the B–N peak corresponding to BN (397.4 eV, Fig. S7a), *m*-BN also shows a new split peak corresponding to the C–N peak at 399.1 eV (Fig. S7b), further proving that benzidine is successfully grafted on the surface of BN. Figure S7c shows the thermogravimetric analyses (TGA) curves of BN and *m*-BN. The weight of BN does not change significantly in the range of 40–800 °C, while the weight loss of *m*-BN increases with increasing temperature. When the temperature rises to 800 °C, the weight loss rate of *m*-BN is about 2.6 wt%, due to the oxidation decomposition of benzidine. As can be seen from Fig. 2b, both BN and *m*-BN present XRD diffraction peaks of the same size, corresponding to 26.6° (002) and 55.2° (004) [52–54]. The morphology (Fig. S8) of the *m*-BN is basically unchanged, compared with that of BN. Results show that “high-temperature solid-phase & diazonium salt decomposition” method does not destroy the crystal structure of BN.

Figure 2c shows XPS spectra of PNF paper, BN/PNF and *m*-BN/PNF nanocomposite paper. The surface of PNF paper mainly contains C, N and O elements. Compared to PNF paper, BN/PNF and *m*-BN/PNF nanocomposite paper shows new B elements attributed to the introduction of BN or *m*-BN. C 1*s* spectra in Fig. 2d–f demonstrate the existence of abundant polar C–N and C–O groups for PNF paper, BN/PNF and *m*-BN/PNF nanocomposite paper. Notably, the characteristic peaks at 285.2, 286.4 and 287.6 eV for the C–N, C–O and N=C–O groups of PNF paper, BN/PNF nanocomposite paper shift to a higher binding energy of 285.8, 286.9 and 288.3 eV for *m*-BN/PNF nanocomposite paper, respectively. Results indicate that the chemical environments for C–N, C–O and N=C–O groups of *m*-BN/PNF nanocomposite paper have been changed, demonstrating the formation of hydrogen-bonding interaction between *m*-BN and PNF.

### Thermal Properties of *m*-BN/PNF Nanocomposite Paper

**Fig. 3:**
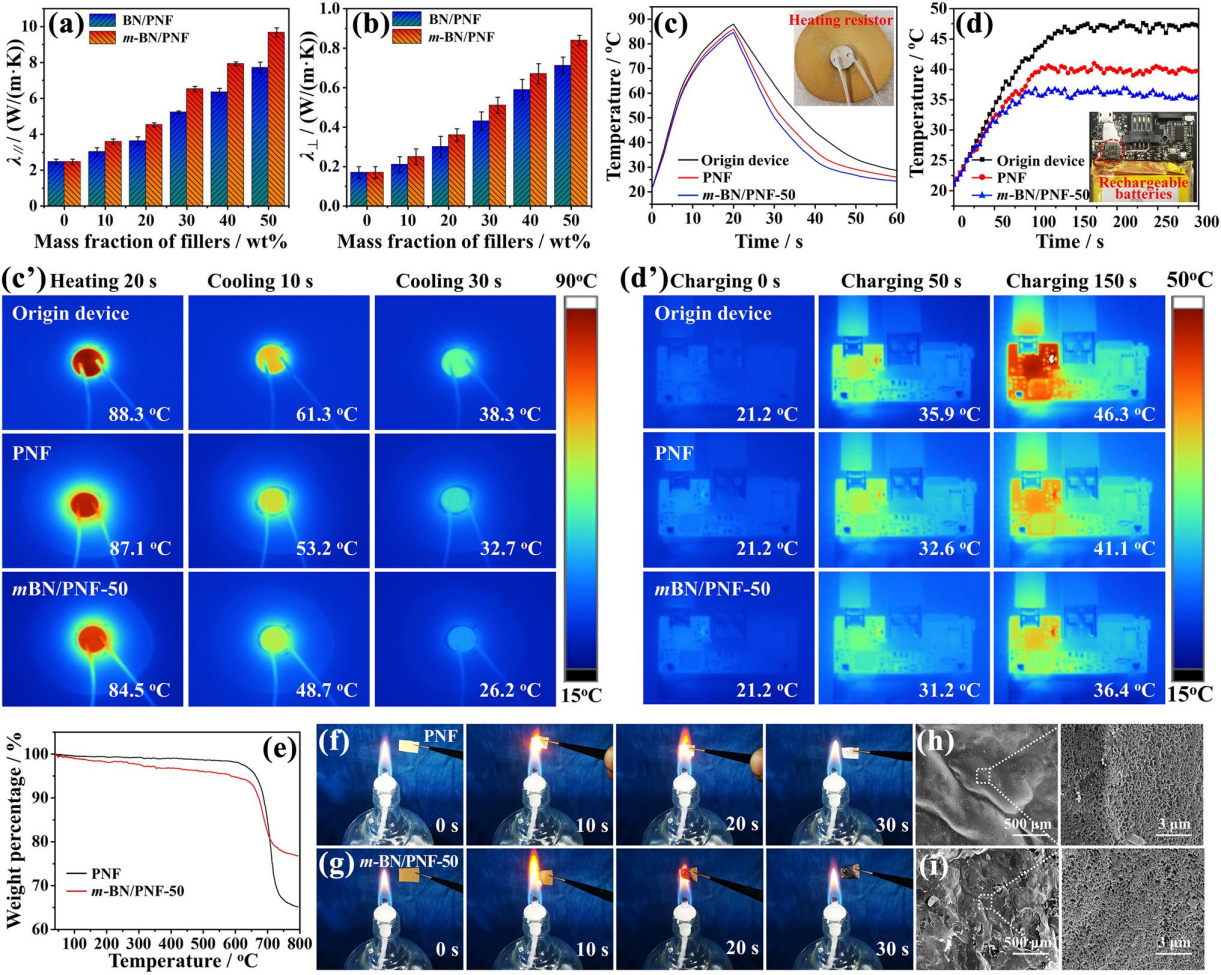
*λ*_∥_ (**a**) and *λ*_⊥_ (**b**) of BN/PNF and *m*-BN/PNF nanocomposite paper at room temperature; the curves of temperatures *vs.* time (**c**) for heating resistor on air, PNF paper and *m*-BN/PNF-50 nanocomposite paper and corresponding infrared thermal images (**c′**); the curves of temperatures *vs.* time (**d**) for the bare lithium-ion rechargeable battery, the lithium-ion rechargeable battery integrated with PNF paper and *m*-BN/PNF-50 nanocomposite paper, and corresponding infrared thermal images (**d′**); TGA curves (**e**) of PNF paper and *m*-BN/PNF-50 nanocomposite paper; optical photographs of PNF paper (**f**) and *m*-BN/PNF-50 nanocomposite paper (**g**) before and during burning; SEM images of PNF paper (**h**) and *m*-BN/PNF-50 nanocomposite paper (**i**) after burning

**Fig. 4:**
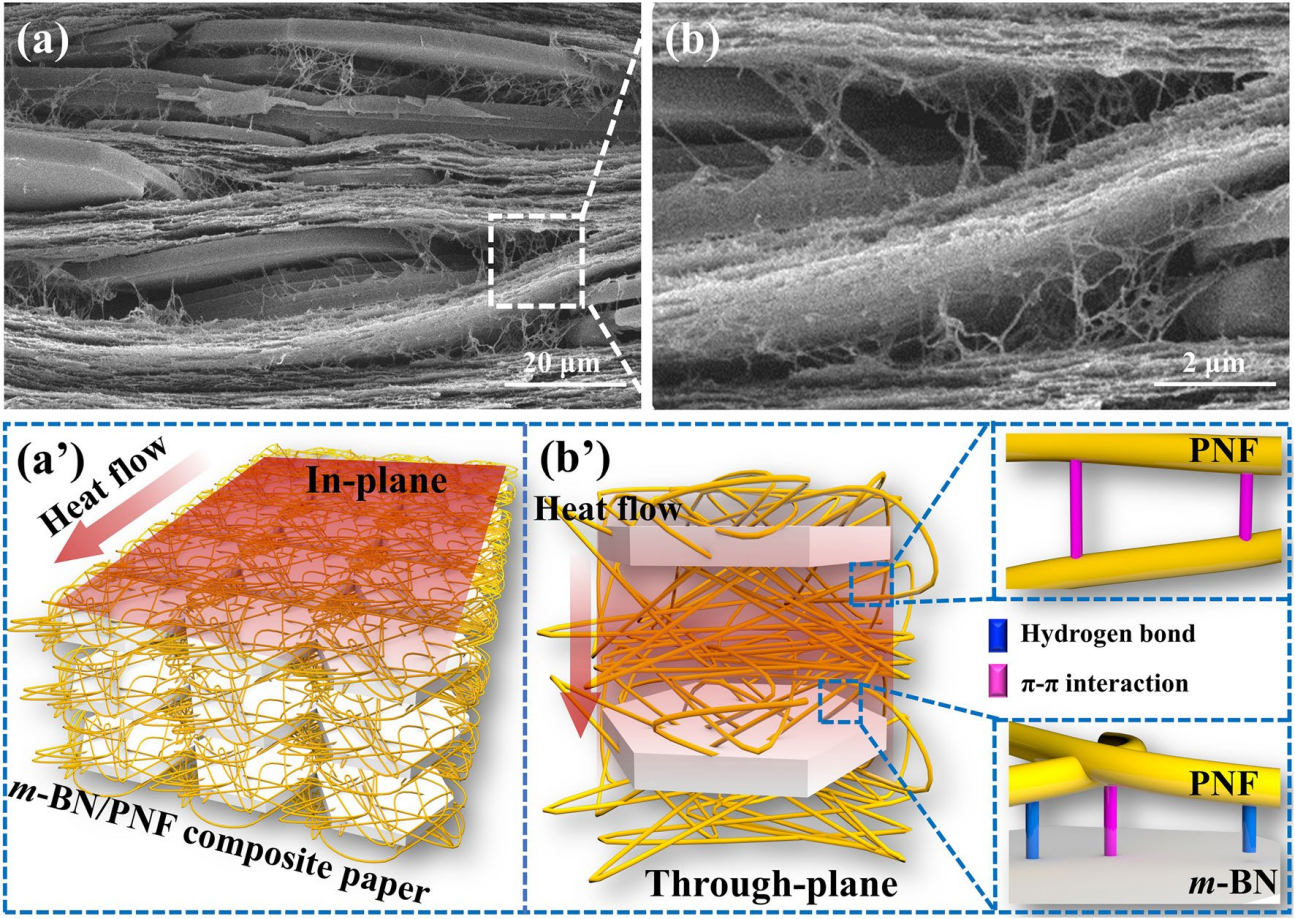
Cross-sectional SEM images (**a**, **b**) of *m*-BN/PNF-50 nanocomposite paper; schematic diagram of thermal conduction for *m*-BN/PNF-50 nanocomposite paper in the in-plane (**a′**) and through-plane (**b′**) direction

Figure 3a, b shows the *λ*_∥_ and through-plane thermal conductivity coefficient (*λ*_⊥_, b) of *m*-BN/PNF nanocomposite paper, respectively. The *λ*_∥_ and *λ*_⊥_ of *m*-BN/PNF and BN/PNF nanocomposite paper all increase with the increasing amount of *m*-BN and BN, mainly due to the fact that the formation probability of thermal conduction pathway increases gradually inner PNF matrix, and the thermal resistance of heat conduction along thermal conduction pathway decreases gradually [55]. Under the same amount of *m*-BN and BN, the *λ*_∥_ and *λ*_⊥_ of the *m*-BN/PNF nanocomposite paper are both higher than those of the BN/PNF nanocomposite paper. When the mass fraction of *m*-BN is 50 wt%, *m*-BN/PNF-50 nanocomposite paper presents the highest *λ*_∥_ and *λ*_⊥_ of 9.68 and 0.84 W m^−1^ K^−1^, respectively, increased by 393% and 494% compared with those of PNF paper, 25.4% and 18.3% higher than the *λ*_∥_ (7.72 W m^−1^ K^−1^) and *λ*_⊥_ (0.71 W m^−1^ K^−1^) of the BN/PNF nanocomposite paper with the same amount of BN. It can be indicated that the *m*-BN presents significant influences on improving the thermal conductivities. This is because the amino groups and biphenyls on the surface of *m*-BN form strong hydrogen bond and π–π interaction with PNF, resulting in the lower interfacial thermal resistance of nanocomposite paper. The *m*-BN is uniformly arranged in the in-plane direction of the *m*-BN/PNF nanocomposite paper (Fig. 4a), which can form efficient *m*-BN thermal conduction pathway (Fig. 4a′). Besides, the *m*-BN is interconnected with a large number of PNF in the through-plane direction (Fig. 4b), which effectively reduces the interfacial thermal resistance between *m*-BN, and drastically improves the phonon propagation efficiency (Fig. 4b′). In contrast, BN is partially agglomerated in the PNF matrix, which cannot form efficient BN thermal conduction pathway, and inevitably introduce the interfacial thermal resistance of BN-BN or BN-PNF [47, 56]. Therefore, BN/PNF nanocomposite paper presents relatively lower *λ*_∥_ and *λ*_⊥_ than those of *m*-BN/PNF nanocomposite paper with the same amount of fillers.

To further elucidate the effect of surface functionalization for BN on the interfacial thermal resistance and *λ* of the *m*-BN/PNF nanocomposite paper, the experimentally obtained *λ*_∥_ and *λ*_⊥_ of BN/PNF and *m*-BN/PNF nanocomposite paper are fitted by the modified Hashin–Shtrikman model [57–59] (Fig. S9). The in-plane thermal resistance ($R_{{\text{c}}}^{*}$) and through-plane $R_{{\text{c}}}^{*}$ of *m*-BN/PNF nanocomposite paper are 0.2336 and 0.2258, respectively, lower than those of BN/PNF nanocomposite paper (in-plane $R_{{\text{c}}}^{*}$ of 0.2443 and through-plane $R_{{\text{c}}}^{*}$ of 0.2317), which further demonstrates that the reduction of interfacial thermal resistance plays a crucial role in improving the thermal conductivity of the nanocomposite paper. Figure S10 shows the *λ*_∥_ and *λ*_⊥_ of PNF paper and *m*-BN/PNF-50 nanocomposite paper at different temperature (20–200 °C). The *λ*_∥_ and *λ*_⊥_ of PNF paper and *m*-BN/PNF-50 nanocomposite paper all increase slightly with the increasing temperature. This is because as the temperature increases, the phonon transmission speed increases, contributing to the improvement of thermal conductivities [60].

Figure 3c, c′ shows the curves of temperatures *vs.* time for heating resistor on air, PNF paper and *m*-BN/PNF-50 nanocomposite paper, and corresponding infrared thermal images. The surface temperature of the heating resistor rises sharply to about 90 °C after 20 s of operation at 10 V. Subsequently, the surface temperature gradually decreases after the heating is stopped, and eventually decreases to below 40 °C. When the air and PNF paper are used as the heat dissipation materials, the surface temperatures of the heating resistor after heating 20 s are 88.3 and 87.1 °C, respectively, higher than the surface temperature of 84.5 °C when *m*-BN/PNF-50 nanocomposite paper is used as the heat dissipation material. The main reason is that *m*-BN/PNF nanocomposite paper can effectively dissipate part of the accumulated heat during heating, due to its excellent thermal conductivity. After cooling for 30 s, the surface temperature of the heating resistance with *m*-BN/PNF-50 nanocomposite paper as the heat dissipation material is as low as 26.2 °C, much lower than those of air (32.7 °C) or PNF paper (38.3 °C).

The *m*-BN/PNF-50 nanocomposite paper is also used as the heat dissipation material in the lithium-ion rechargeable battery (Fig. S11). Figure 3d, d′ shows the curves of temperatures *vs.* time for the bare lithium-ion rechargeable battery, the lithium-ion rechargeable battery integrated with PNF paper and *m*-BN/PNF-50 nanocomposite paper and the corresponding infrared thermal images. When the lithium-ion rechargeable battery starts to work, the temperature of the core components increases gradually, among which the bare core components show the highest heating rate, and that of the *m*-BN/PNF-50 nanocomposite paper shows the lowest. After charging for 150 s, the surface temperature of core components integrated with *m*-BN/PNF-50 nanocomposite paper stabilizes around 36.4 °C, significantly lower than the surface temperature of bare core component of 46.3 °C and core components integrated with PNF paper of 41.1 °C. This is mainly because the *m*-BN/PNF-50 nanocomposite paper presents excellent heat dissipation property, which can quickly diffuse the heat from the lithium-ion rechargeable battery. Therefore, the *m*-BN/PNF-50 nanocomposite paper is an efficient kind of thermal management material with broad application prospects in lithium batteries and integrated circuits.

Figure 3e shows TGA curves of PNF paper and *m*-BN/PNF-50 nanocomposite paper. PNF paper and *m*-BN/PNF-50 nanocomposite paper exhibit excellent heat resistances, and *m*-BN/PNF-50 nanocomposite paper has only slight weight loss below 640 °C, attributed to the degradation of benzidine. When the temperature is higher than 640 °C, the weight of PNF paper and *m*-BN/PNF-50 nanocomposite paper begins to decrease significantly, mainly attributed to the carbonization of PBO molecular chains. Figure 3f–i shows optical photographs of PNF paper (f) and *m*-BN/PNF-50 nanocomposite paper (g) before and during burning, SEM images of PNF paper (h) and *m*-BN/PNF-50 nanocomposite paper (i) after burning, respectively. PNF paper and *m*-BN/PNF-50 nanocomposite paper only shrink and curl slightly when they stay on the flame for 10 s. After removing the flame (> 20 s), no obvious flame and smoke are observed on PNF paper and *m*-BN/PNF-50 nanocomposite paper, and they basically maintain the original shape. From Fig. 3h, i, PNF paper and *m*-BN/PNF-50 nanocomposite paper form a dense carbon layer on the surface after burning, which can prevent oxygen from entering the interior [61, 62], showing that PNF paper and *m*-BN/PNF-50 nanocomposite paper both have excellent flame-retardant properties. To further evaluate the flame-retardant properties of PNF paper and *m*-BN/PNF-50 nanocomposite paper, The microscale combustion calorimetry is used to quantificationally measure the heat release rate of PNF paper and *m*-BN/PNF-50 nanocomposite paper (Fig. S12). The *m*-BN/PNF-50 nanocomposite paper exhibits the low peak heat release rate of 154.3 W g^−1^, lower than that of the PNF paper (221.4 W g^−1^). Results suggest that the introduction of *m*-BN further improves the flame-retardant property of *m*-BN/PNF-50 nanocomposite paper. The reason is that *m*-BN presents extremely excellent thermal stability (Fig. S7c) and can be used as a flame retardant to prevent the spread of flame, thereby improving the flame-retardant property.

### Electrical Insulation of *m*-BN/PNF Nanocomposite Paper

**Fig. 5:**
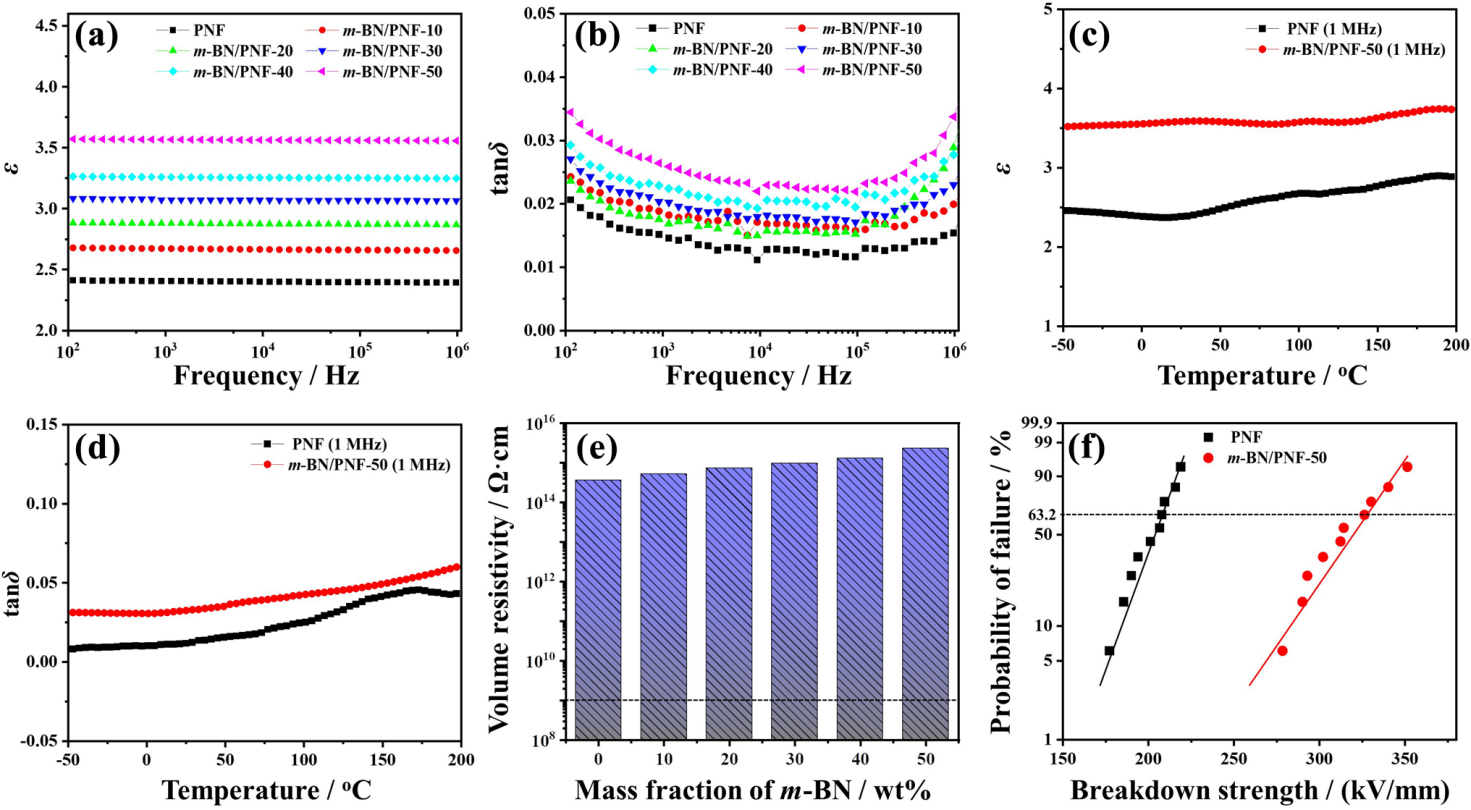
*ε* (**a**) and tan*δ* (**b**) of *m*-BN/PNF nanocomposite paper at different frequencies; *ε* (**c**) and tan*δ* (**d**) of PNF paper and *m*-BN/PNF-50 nanocomposite paper in the range of − 50 to 200 °C; volume resistivity (**e**) of *m*-BN/PNF nanocomposite paper; Weibull plots for breakdown strength (**f**) of PNF paper and *m*-BN/PNF-50 nanocomposite paper

Figure 5a, b shows the dielectric constant (*ε*) and dielectric loss tangent (tan*δ*) of *m*-BN/PNF nanocomposite paper at different frequency. At the same frequency, *ε* and tan*δ* of *m*-BN/PNF nanocomposite paper gradually increase with the increasing amount of *m*-BN. When the mass fraction of *m*-BN is 50 wt%, the *ε* and tan*δ* of *m*-BN/PNF-50 nanocomposite paper are 3.55 and 0.033 (1 MHz), higher than that (*ε* of 2.39 and tan*δ* of 0.015, 1 MHz) of PNF paper, but lower than that (*ε* of 3.76 and tan*δ* of 0.042, 1 MHz, Fig. S13) of BN/PNF-50 nanocomposite paper. This is because BN with high *ε* (~ 4.0) increases the *ε* of the nanocomposite paper. In addition, the introduction of *m*-BN or BN produces new interfaces with PNF. Under the action of applied electric field, charge carriers accumulate on the interface and induce interfacial polarization, which further increases the *ε* and tan*δ.* Compared with BN/PNF nanocomposite paper, *m*-BN forms strong hydrogen bond and π–π interaction with PNF, which would reduce the interfacial polarization, resulting in relatively lower *ε* and tan*δ* of *m*-BN/PNF nanocomposite paper.

In addition, the *ε* (3.55–3.59) and tan*δ* (0.021–0.033) of *m*-BN/PNF-50 nanocomposite paper show no significant changes in the range of 100 Hz–1 MHz, indicating excellent frequency stability. Figure 5c, d shows the *ε* and tan*δ* of PNF paper and *m*-BN/PNF-50 nanocomposite paper in the range of − 50 to 200 °C. The *ε* and tan*δ* of PNF paper and *m*-BN/PNF-50 nanocomposite paper generally increase with increasing temperature. When the temperature rises from − 50 to 200 °C, the *ε* of *m*-BN/PNF-50 nanocomposite paper increases from 3.51 to 3.73, showing an increase of 0.22, lower than the increase (0.43) of PNF paper. The tan*δ* increases from 0.031 to 0.059, showing an increase of 0.028, lower than the increase (0.035) of PNF paper. The main reason is that, within a certain range, the increase of temperature is conducive to the orientation of molecular chains, promoting the generation of atomic and orientation polarization [63, 64], which leads to the increasing *ε* and tan*δ* of the PNF paper and *m*-BN/PNF nanocomposite paper. While BN presents excellent thermal stability, and its internal molecular structure is difficult to change in a wide temperature range, so that the *ε* and tan*δ* are less affected by temperature. Overall, the dielectric properties of *m*-BN/PNF nanocomposite paper exhibit better temperature stability than that of PNF paper.

Figure 5e shows volume resistivity of *m*-BN/PNF nanocomposite paper. Volume resistivity of *m*-BN/PNF nanocomposite paper increases with the increasing amount of *m*-BN. The *m*-BN/PNF-50 nanocomposite paper has the highest volume resistivity of 2.3 × 10^15^ Ω cm, higher than that of the PNF paper (3.6 × 10^14^ Ω cm), which meets the requirements for the use of insulating materials inside electronic/electrical devices (> 10^9^ Ω cm) [65]. To our satisfaction, the *m*-BN/PNF-50 nanocomposite paper presents high breakdown strength (Fig. 5f) of 324.2 kV mm^−1^. The excellent electrical insulation is attributed to the high volume resistivity (10^16^–10^18^ Ω cm) of *m*-BN, and charged carriers are difficult to carry out multiple migration between *m*-BN and PNF, promoting the high volume resistivity of *m*-BN/PNF-50 nanocomposite paper. Under the action of applied voltage, *m*-BN can be used as the scattering point, and ejected electrons in the PNF matrix directly collide with *m*-BN and lose energy [66–68]. The *m*-BN/PNF-50 nanocomposite paper requires higher voltage to achieve breakdown.

### Mechanical Properties of *m*-BN/PNF Nanocomposite Paper

**Fig. 6:**
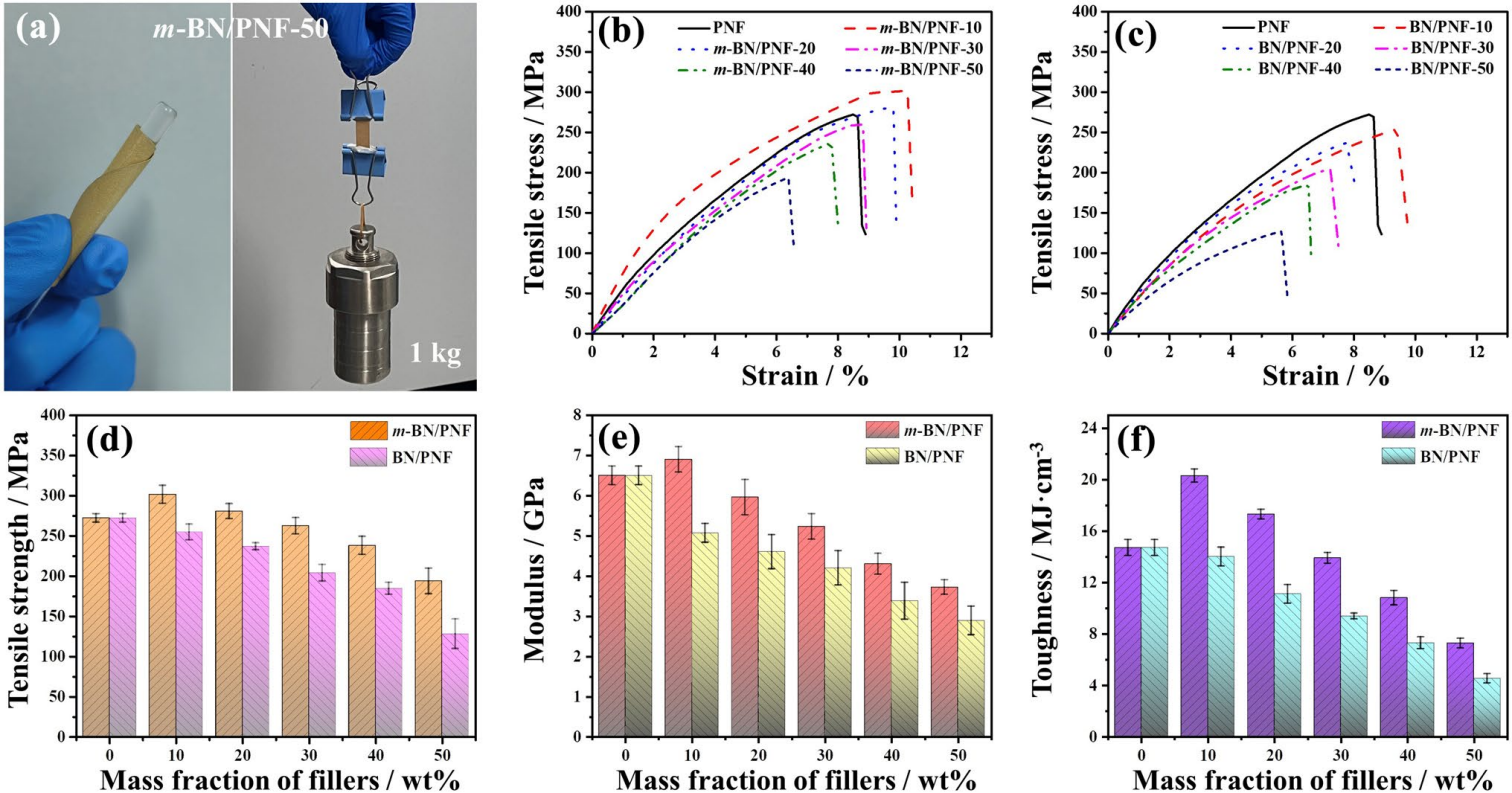
Optical photographs of *m*-BN/PNF-50 nanocomposite paper possessing ultra-flexibility and withstanding a 1-kg reactor (**a**); tensile stress–strain curves of BN/PNF (**b**) and *m*-BN/PNF (**c**) nanocomposite paper; tensile strength (**d**), tensile modulus (**e**) and toughness (**f**) of BN/PNF and *m*-BN/PNF nanocomposite paper

Figure 6a shows that the* m*-BN/PNF-50 nanocomposite paper can be bent arbitrarily, and withstand a 1-kg reactor without any crack or fracture, indicating flexibility and robust mechanical properties. From Fig. 6b, c, after linear elastic deformation and yielding, BN/PNF and *m*-BN/PNF nanocomposite paper undergoes obvious plastic elongation until fracture. Figure 6d–f shows tensile strength (d), tensile modulus (e) and toughness (f) of BN/PNF and *m*-BN/PNF nanocomposite paper. With the increasing amount of *m*-BN and BN, the tensile strength, tensile modulus and toughness of *m*-BN/PNF-50 nanocomposite paper increase first and then decrease, while the tensile strength, tensile modulus and toughness of BN/PNF-50 nanocomposite paper gradually decrease. Under the same amount of *m*-BN and BN, the tensile strength, tensile modulus and toughness of *m*-BN/PNF nanocomposite paper are all higher than those of the BN/PNF nanocomposite paper. When the mass fraction of *m*-BN is 10 wt%, the tensile strength and modulus and toughness of the *m*-BN/PNF-10 nanocomposite paper reach the maximum values of 301.5 MPa, 6.9 GPa and 20.3 MJ m^−3^, respectively, 10.8%, 6.2% and 38.1% higher than those of PNF paper, also higher than the tensile strength (254.6 MPa), tensile modulus (5.1 GPa) and toughness (14.0 MJ m^−3^) of BN/PNF-10 nanocomposite paper. When the mass fraction of *m*-BN is 50 wt%, the tensile strength (193.6 MPa), tensile modulus (3.72 GPa) and toughness (7.26 MJ m^−3^) of *m*-BN/PNF-50 nanocomposite paper are slightly lower than those of PNF paper.

The excellent mechanical properties are mainly attributed to the construction of extensive hydrogen bonds and π–π interactions between *m*-BN and PNF, and stable nacre-mimetic layered structures. The introduction of appropriate amount of *m*-BN can effectively slow down the crack propagation and transfer stress and improve the mechanical properties of *m*-BN/PNF nanocomposite paper. However, excessive *m*-BN tends to form stress concentration points in the *m*-BN/PNF nanocomposite paper. In contrast, the relatively poor compatibility between BN and PNF leads to the formation of more defects and stress concentration points within the BN/PNF nanocomposite paper, resulting in the serious decline in mechanical properties.

As shown in Table S1, *m*-BN/PNF-50 nanocomposite paper presents optimal thermal stability (thermal decomposition temperature up to 640 °C) and excellent thermal conductivity among the reported electrically insulating paper. Meanwhile, *m*-BN/PNF nanocomposite paper has excellent mechanical properties and high breakdown strength, showing a broad application prospect in high-end thermal management fields such as electronic devices and electrical equipment.

## Conclusions

FTIR, XRD and TGA show that benzidine has been successfully grafted on the BN surface (*m*-BN). TEM and SEM indicate that PBO fibers are exfoliated into PNF, and *m*-BN and PNF have formed 3D crosslinked network structures. When the mass fraction of *m*-BN is 50 wt%, *m*-BN/PNF-50 nanocomposite paper presents the highest *λ*_∥_ and *λ*_⊥_ of 9.68 and 0.84 W m^−1^ K^−1^, respectively, increased by 393% and 494% compared with the PNF paper. The nanocomposite paper also presents excellent electrical insulation (volume resistivity of about 2.3 × 10^15^ Ω cm and breakdown strength of 324.2 kV mm^−1^), and its dielectric properties exhibit excellent frequency and temperature stability. In addition, the nanocomposite paper has excellent mechanical properties (tensile strength of 193.6 MPa), outstanding thermal stability (thermal decomposition temperature > 640 °C) and flame retardancy (self-extinguishing immediately after evacuation from the flame).

## Supplementary Information

Below is the link to the electronic supplementary material.

Supplementary file1 (PDF 1215 kb)
