# Supplementary material for: Flexible and Robust Functionalized Boron Nitride/Poly(p-Phenylene Benzobisoxazole) Nanocomposite Paper with High Thermal Conductivity and Outstanding Electrical Insulation
Source: Nanomicro Lett. 2023 Nov 30;16:38. doi: 10.1007/s40820-023-01257-5 (PMC10689708; doi:10.1007/s40820-023-01257-5)
Supplement: Supplementary file 1 — Supplementary file1 (PDF 1215 kb) [file 40820_2023_1257_MOESM1_ESM.pdf]

Supporting Information for

Flexible and Robust Functionalized Boron Nitride/Poly(*p*-Phenylene Benzobisoxazole) Nanocomposite Paper with High Thermal Conductivity and Outstanding Electrical Insulation

Lin Tang[1], Kunpeng Ruan[2], Xi Liu[1], Yusheng Tang[2,]*, Yali Zhang[2], Junwei Gu[2,]*

[1] Chongqing Key Laboratory of Green Synthesis and Applications, College of Chemistry, Chongqing Normal University, Chongqing 401331, P. R. China

[2] Shaanxi Key Laboratory of Macromolecular Science and Technology, School of Chemistry and Chemical Engineering, Northwestern Polytechnical University, Xi'an 710072, P. R. China

*Corresponding authors. E-mail: tys@nwpu.edu.cn (Y. Tang); gjw@nwpu.edu.cn or nwpugjw@163.com (J. Gu).

S1 Main Raw Materials

Poly(*p*-phenylene-2,6-benzobisoxazole) (PBO) fibers, with a density of 1.56 g/cm^3 and the trade name Zylon HMPBO, were purchased from Toyobo Co. Ltd. (Osaka, Japan). Boron nitride, with the particle size of 5~10 μm and thickness of 100~500 nm, was received from Zhejiang Yamei Nano Technology Co., Ltd (Jiaxing, China). Sodium sulfate (Na_2SO_4, 99%) was purchased from Shanghai Aladdin Biochemical Co., Ltd. (Shanghai, China). Trifluoroacetic acid (99%), methanesulfonic acid (99%), sodium hydroxide (97%), potassium hydroxide (97%), sodium nitrite (99%) and iron powder (99.5%) were all received from Shanghai Macklin Biochemical Co., Ltd. (Shanghai, China). Benzidine (98%) was received from Shanghai Bailiwick Chemical Technology Co., Ltd (Shanghai, China). Hydrochloric acid (37%) was purchased from Beijing Chemical Works (Beijing, China). Methanol (99.85%) was purchased from Shanghai Sigma Aldrich Trading Co., Ltd. (Shanghai, China).

S2 Characterizations

Fourier transform infrared (FT-IR) spectra of the samples were obtained on Bruker Tensor 27 equipment (Bruker Corp., Germany). X-ray photoelectron spectroscopy (XPS) analyses of the samples were carried out by Kratos Axis Ultra DLD equipment (Kratos Corp., UK). X-ray diffraction (XRD) spectra of the samples were carried out on a Shimadzu-7000 type X-ray diffraction (Shimadzu, Japan). Thermogravimetric analyses (TGA) of the samples were carried out by STA 449F3 (NETZSCH C Corp., Germany) at 10°C/min (argon atmosphere), over the whole range of temperature (40~800°C). Transmission electron microscope (TEM) images of the samples were collected on a Talos F200X/TEM microscope (FEI Company, USA). Scanning electron microscope (SEM) morphologies of the samples were observed using a VEGA3-LMH (TESCAN Corp., Czech Republic). In-plane thermal conductivity coefficient ($\lambda_{//}$) and through-plane thermal conductivity coefficient ($\lambda_{\perp}$) of the samples were characterized with Hot Disk TPS2200 thermal constant analyzer (AB Co., Sweden). Infrared thermal images of the samples were obtained by Ti 300 infrared thermography (Fluke Co.,

USA). The microscale combustion calorimetry (MCC) was based on an MCC-2 microscale combustion calorimeter (FTT Company, UK) with a heating rate of 1ºC/s in an air atmosphere, over the whole range of temperature (20~700°C). The volume resistivity of the samples was measured by high resistance meter tester according to the standard of IEC 60243-1: 2013. The breakdown strength of the samples was measured *via* an MPD-104 high-voltage generator (Partulab Technology Co. Ltd, China). Dielectric properties of the samples were measured by WK6500B precision impedance analyzer (Wayne Kerr Electronics Corp., UK). Dielectric properties of the samples at -50~200°C were measured by Agilent4294A precision impedance analyzer (Agilent Technologies Inc., USA). Tensile properties of the samples were measured by a tensile testing machine (Instron Co., USA) according to ASTM D5568-08.

S3 Supplementary Figures

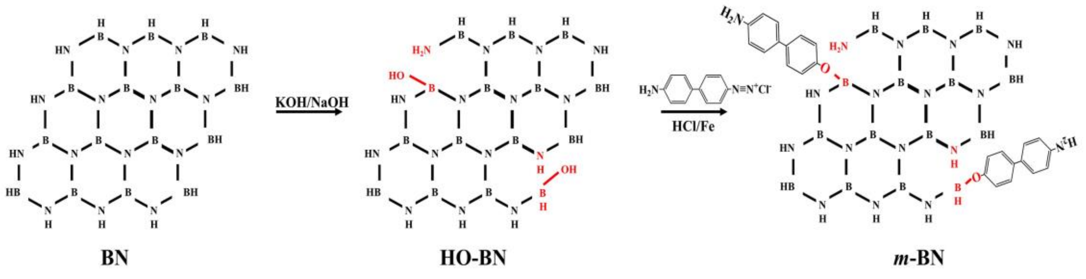

Fig. S1 Schematic diagram of the preparation for *m*-BN

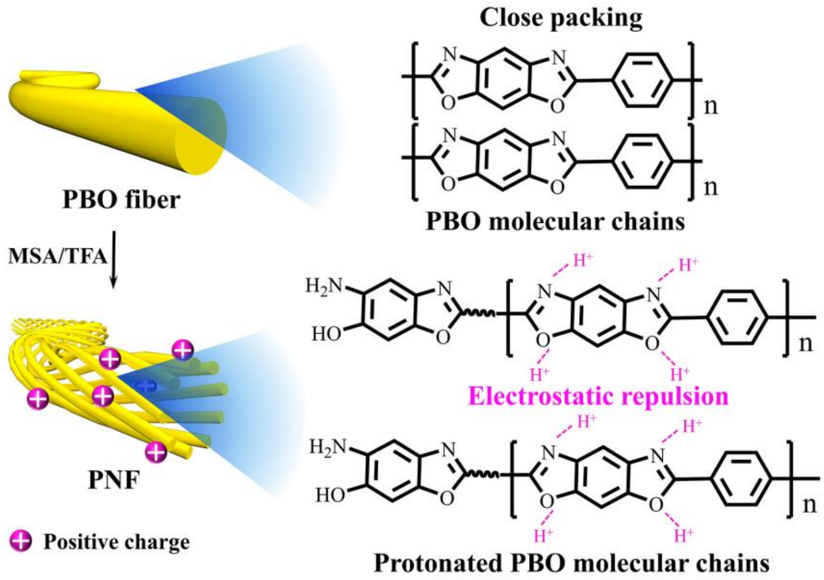

Fig. S2 Schematic diagram of the exfoliation for the PBO fiber into PNF

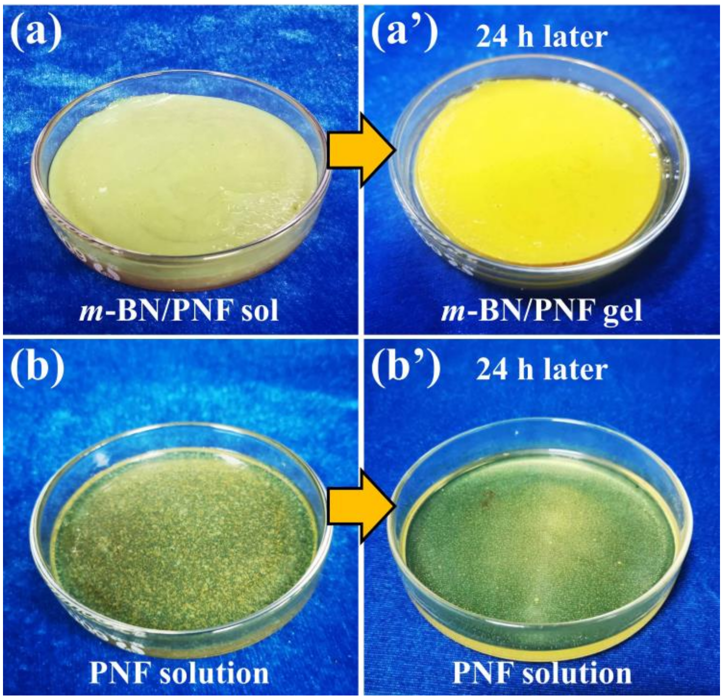

Fig. S3 Optical photographs of *m*-BN sol left to form *m*-BN gel for 24 h (**a, a'**); optical photographs of PNF solution after being left for 24 h (**b, b'**)

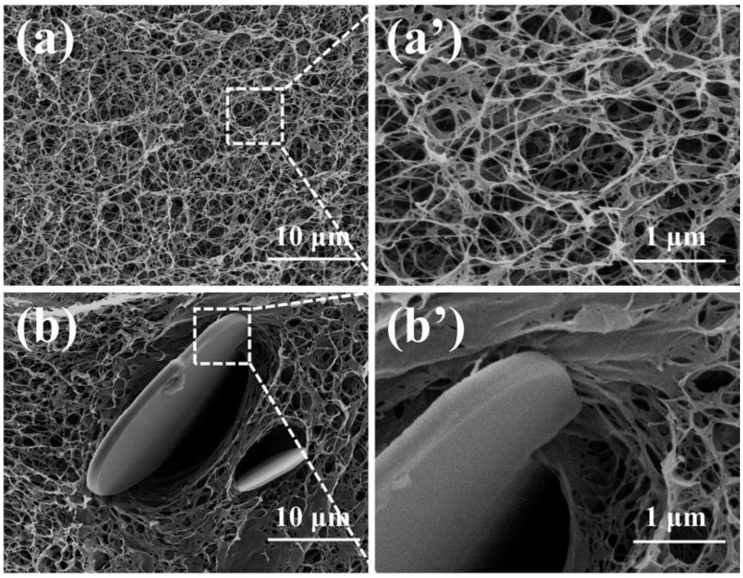

Fig. S4 SEM images showing the inside of the PNF (**a, a'**) and BN/PNF gels (**b, b'**)

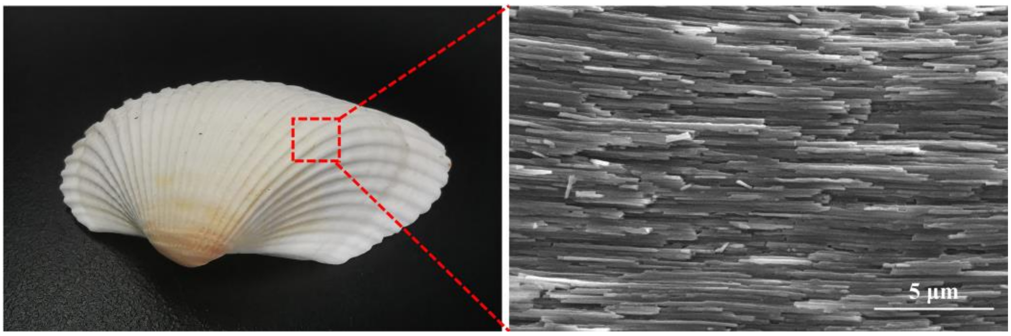

Fig. S5 Optical and SEM images of the conch

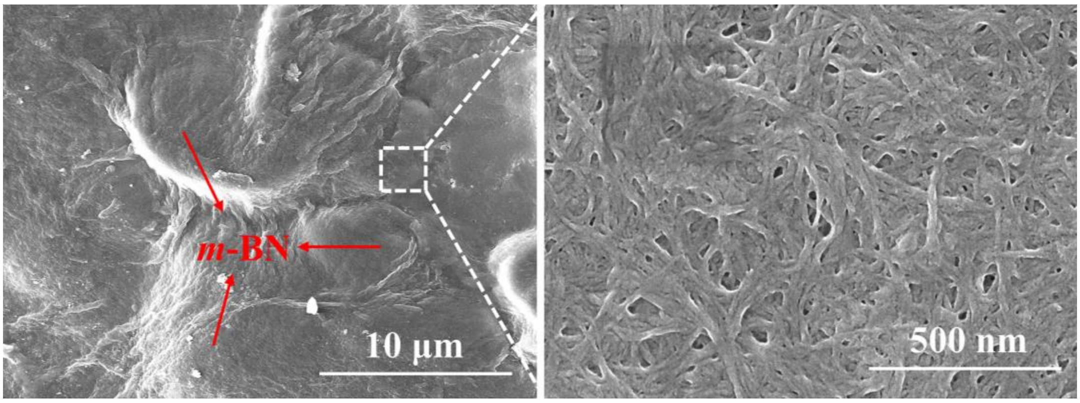

Fig. S6 SEM images of the surface for *m*-BN/PNF nanocomposite paper

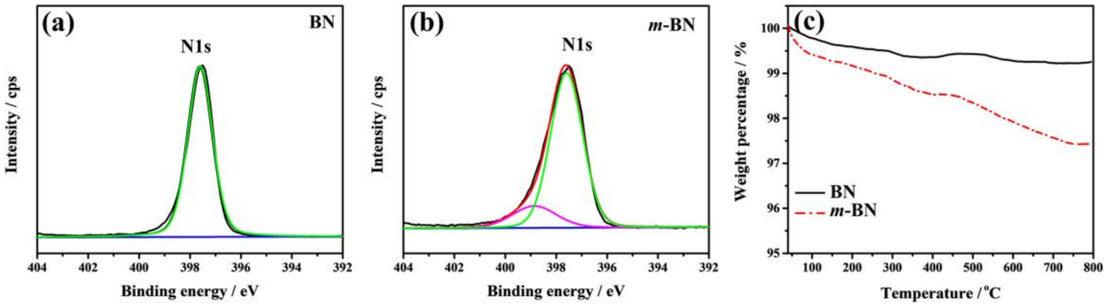

Fig. S7 High-resolution N 1s XPS spectra of BN (**a**) and *m*-BN (**b**); TGA curves of BN and *m*-BN (**c**)

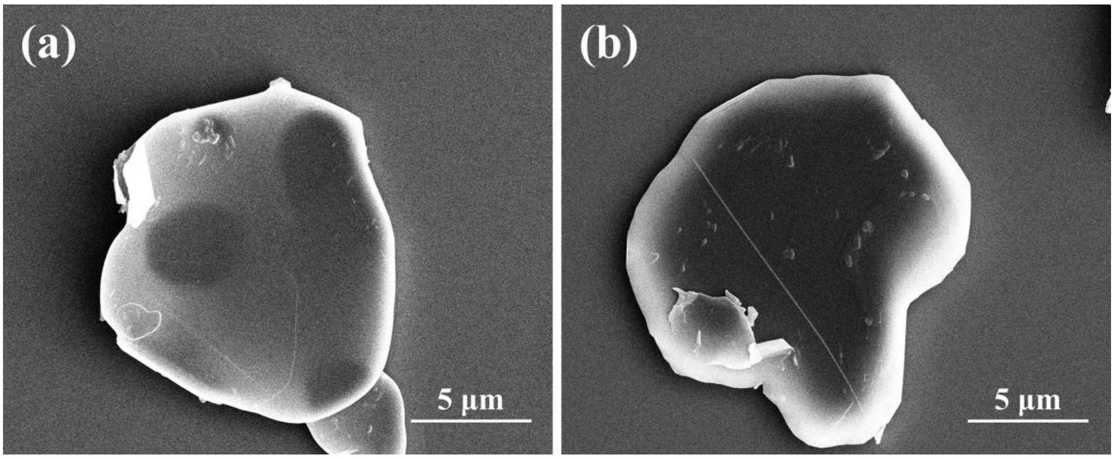

Fig. S8 SEM images of BN (**a**) and *m*-BN (**b**)

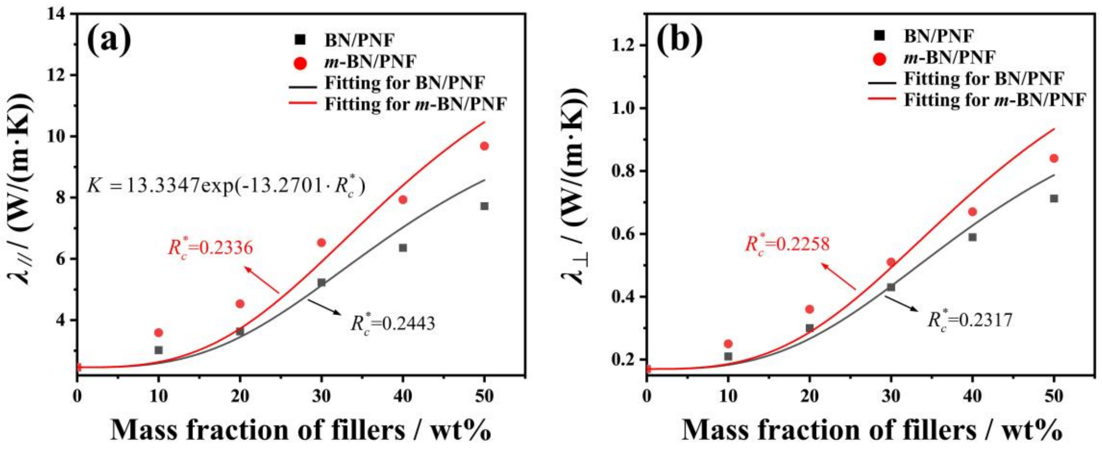

Fig. S9 Fitting $\lambda_{//}$ (**a**) and $\lambda_{\perp}$ (**b**) of BN/PNF and *m*-BN/PNF nanocomposite paper by modified Hashin-Shtrikman model

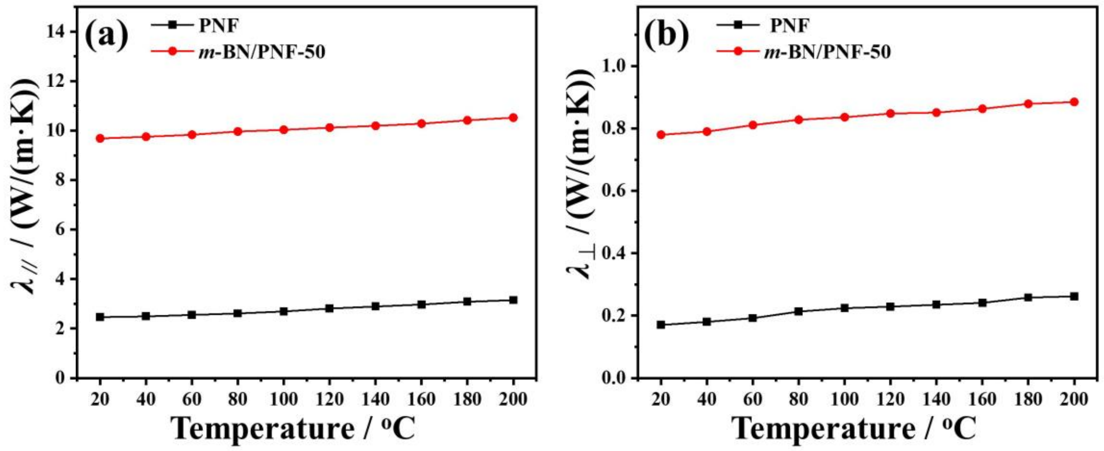

Fig. S10 $\lambda_{//}$ (**a**) and $\lambda_{\perp}$ (**b**) of PNF paper and *m*-BN/PNF-50 nanocomposite paper at different temperature

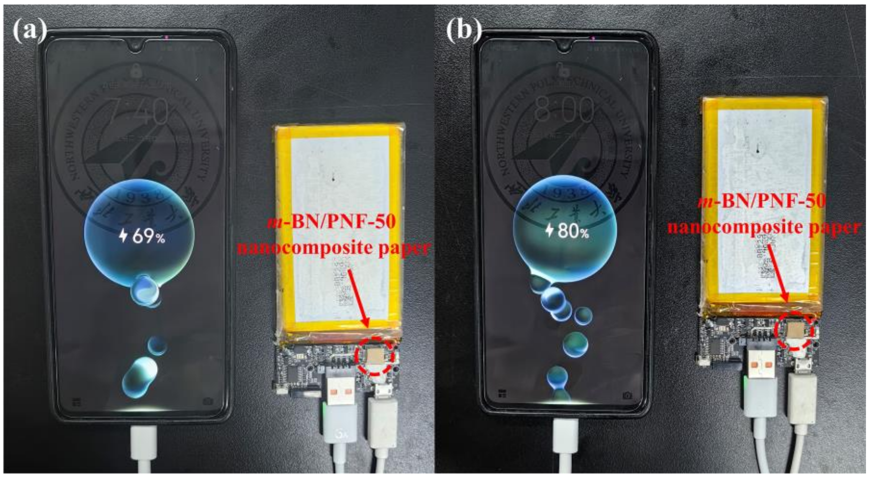

Fig. S11 The process of charging the mobile phone with the lithium-ion rechargeable battery

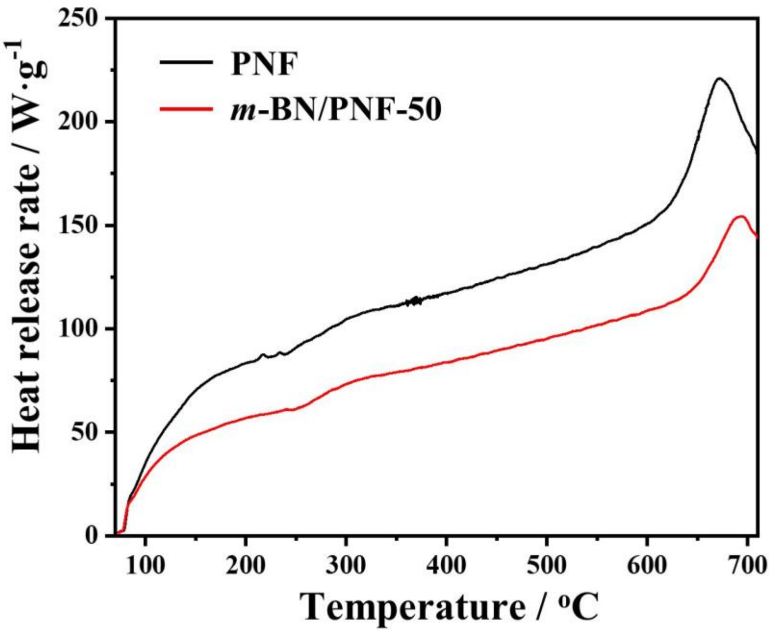

Fig. S12 Heat release rate curves of PNF paper and *m*-BN/PNF-50 nanocomposite paper

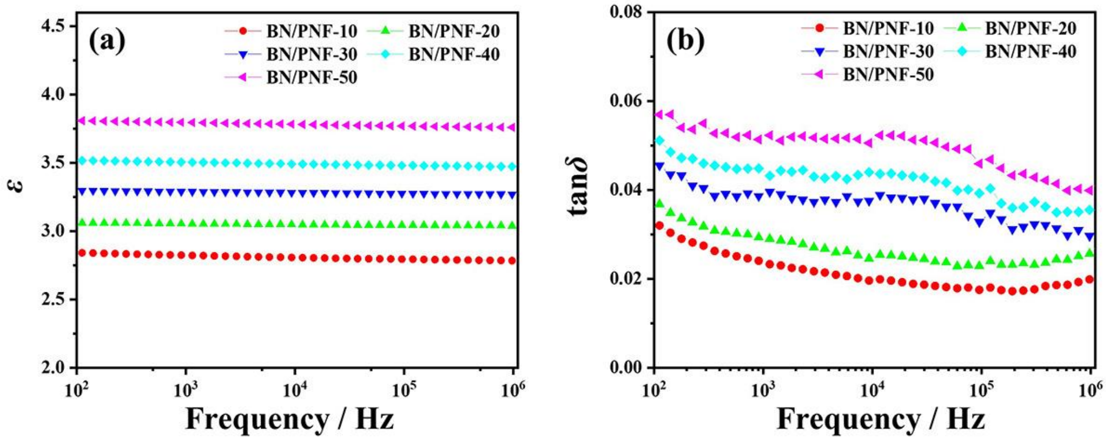

Fig. S13 ε (a) and tanδ (b) of BN/PNF nanocomposite paper at room temperature

S4 Supplementary Table

Table S1 Comparison of the properties of *m*-BN/PNF-50 nanocomposite paper with those of other reported electrical insulating paper

Materials	λ / W/(m·K)	Breakdown strength / kV/mm	Tensile strength / MPa	Thermal decomposition temperature / °C	Refs.
RC/BTNF composite film	--	370.0	110.0	~330	[S1]
RC/PDA@BTNF composite film	--	515.0	129.0	~340	[S2]
BNNS/CNF aerogel nano-paper	2.40	--	--	293	[S3]
BN/MoS_2/PCNF composite film	2.30	--	55.5	~300	[S4]
h-BN/CNF composite film	1.50	--	--	292	[S5]
CNF/GNP-g-L/D composite film	9.36	--	82.5	350	[S6]
ANF-NFC/mica@PDA composite film	--	33.5	28.8	231	[S7]
Hot-pressed PPTA nanopaper	--	92.8	159.6	564	[S8]
f-BNNS/s-ANFs composite paper	0.224	93.5	50.5	--	[S9]
aBNN/ANF nanocomposite film	4.61	90.0	175.0	538	[S10]
BNNSs/ANF composite insulating paper	4.34	59.6	64.0	522	[S11]
h-BN@AgNPs/ANFs composite paper	1.03	--	42.0	--	[S12]
ANF/chitosan/Al_2O_3 composite film	--	279.2	232.0	575	[S13]
ANF/MTM nanocomposite film	--	77.2	126.5	--	[S14]
HAP/ANF nanocomposite paper	--	92.4	73.5	500	[S15]
NTS/ANF-X 40/60 nanopaper	--	164.0	175.0	565	[S16]
m-BN/PNF-50 nanocomposite paper	9.68	324.2	193.6	640	This work

Supplementary References

[S1] C. Zhang, Y. Yin, Q. Yang, Z. Shi, G.-H. Hu, C. Xiong. Flexible cellulose/batio3 nanocomposites with high energy density for film dielectric capacitor. ACS Sustainable Chem. Engin. **7**(12), 10641-10648 (2019). https://doi.org/10.1021/acssuschemeng.9b01302

[S2] Y. Yin, C. Zhang, J. Chen, W. Yu, Z. Shi, C. Xiong, Q. Yang. Cellulose/$BaTiO_3$ nanofiber dielectric films with enhanced energy density by interface modification with poly(dopamine). Carbohydrate Polymers **249**, 116883 (2020). https://doi.org/https://doi.org/10.1016/j.carbpol.2020.116883

[S3] X. Wang, Z. Yu, H. Bian, W. Wu, H. Xiao, H. Dai. Thermally conductive and electrical insulation BnNs/CNF aerogel nano-paper. Polymers **11**(4), 660 (2019). https://doi.org/10.3390/polym11040660

[S4] L. Yang, W. Xu, X. Shi, M. Wu, Z. Yan, Q. Zheng, G. Feng, L. Zhang, R. Shao. Investigating the thermal conductivity and flame-retardant properties of Bn/MoS_2/PCNF composite film containing low Bn and MoS_2 nanosheets loading. Carbohydrate Polymers **311**, 120621 (2023). https://doi.org/https://doi.org/10.1016/j.carbpol.2023.120621

[S5] X. Wang, Z. Yu, L. Jiao, H. Bian, W. Yang, W. Wu, H. Xiao, H. Dai. Aerogel perfusion-prepared h-bn/cnf composite film with multiple thermally conductive pathways and high thermal conductivity. Nanomaterials **9**(7), (2019). https://doi.org/10.3390/nano9071051

[S6] T. Gu, D.-x. Sun, X. Xie, X.-d. Qi, J.-h. Yang, C.-s. Zhao, Y.-z. Lei, Y. Wang. Highly thermally conductive, electrically insulated and flexible cellulose nanofiber-based composite films achieved via stereocomplex crystallites cross-linked graphene nanoplatelets. Compos. Sci. Technol. **230**, 109757 (2022). https://doi.org/https://doi.org/10.1016/j.compscitech.2022.109757

[S7] Y. Wang, W. Dang, C. Yao, C. Tian, Z. Lu. Flexible and high-temperature dielectric anf-nfc/mica@pda composite film with high breakdown strength. J. Mater. Sci. **57**(43), 20174-20186 (2022). https://doi.org/10.1007/s10853-022-07878-2

[S8] Z. Lu, L. Si, W. Dang, Y. Zhao. Transparent and mechanically robust poly (para-phenylene terephthamide) ppta nanopaper toward electrical insulation based on nanoscale fibrillated aramid-fibers. Compos. Part A: Appl. Sci. Manufact. **115**, 321-330 (2018). https://doi.org/https://doi.org/10.1016/j.compositesa.2018.10.009

[S9] S. Chen, S. Song, Z. Li, F. Xie, F. Jia, K. Gao, Y. Wang, Z. Lu. Constructing a bnns/aramid nanofiber composite paper via thiol-ene click chemistry for improved thermal conductivity. Mater. Today Commun. **31**, 103806 (2022). https://doi.org/https://doi.org/10.1016/j.mtcomm.2022.103806

[S10] L. Xia, X. Zheng, R. Yang, X. Yuan, M. Jiang, X. Zhuang. Robust nanocomposite films with high dielectric insulation from boron nitride nanosheet and aramid nanofiber. Fibers Polym. **24**(3), 1131-1138 (2023). https://doi.org/10.1007/s12221-023-00109-8

[S11] M. Li, Y. Zhu, C. Teng. Facial fabrication of aramid composite insulating paper with high strength and good thermal conductivity. Compos. Commun. **21**, 100370 (2020). https://doi.org/https://doi.org/10.1016/j.coco.2020.100370

[S12] L. Zhuo, S. Chen, F. Xie, P. Qin, Z. Lu. Toward high thermal conductive aramid nanofiber papers: Incorporating hexagonal boron nitride bridged by silver nanoparticles. Polym. Compos. **42**(4), 1773-1781 (2021). https://doi.org/https://doi.org/10.1002/pc.25932

[S13] Q.-W. Qing, C.-M. Wei, Q.-H. Li, R. Liu, Z.-X. Zhang, J.-W. Ren. Bioinspired dielectric film with superior mechanical properties and ultrahigh electric breakdown strength made from aramid nanofibers and alumina nanoplates. Polymers **13**(18), 3093 (2021). https://doi.org/10.3390/polym13183093

[S14] L. Si, Z. Lu, C. Yao, Q. Ma, Y. Zhao, Y. Wang, D. Wang, Z. Jin. Nacre-like nanocomposite film with excellent dielectric insulation properties and

mechanical strength based on montmorillonite nanosheet and aramid nanofiber. J. Mater. Sci. **55**(14), 5948-5960 (2020). https://doi.org/10.1007/s10853-020-04412-0

[S15] Z.-Y. Wang, Y.-J. Zhu, Y.-Q. Chen, H.-P. Yu, Z.-C. Xiong. Flexible nanocomposite paper with superior fire retardance, mechanical properties and electrical insulation by engineering ultralong hydroxyapatite nanowires and aramid nanofibers. Chem. Engin. J. **444**, 136470 (2022). https://doi.org/https://doi.org/10.1016/j.cej.2022.136470

[S16] F. Zeng, X. Chen, G. Xiao, H. Li, S. Xia, J. Wang. A bioinspired ultratough multifunctional mica-based nanopaper with 3d aramid nanofiber framework as an electrical insulating material. ACS Nano **14**(1), 611-619 (2020). https://doi.org/10.1021/acsnano.9b07192
